# Long-Term Effect of Porcine Brain Enzyme Hydrolysate Intake on Scopolamine-Induced Memory Impairment in Rats

**DOI:** 10.3390/ijms23063361

**Published:** 2022-03-20

**Authors:** Ting Zhang, Min Jung Kim, Min Ju Kim, Xuangao Wu, Hye Jeong Yang, Heng Yuan, Shaokai Huang, Sun Myung Yoon, Keun-Nam Kim, Sunmin Park

**Affiliations:** 1 Department of Bio-Convergence System, Hoseo University, Asan 31499, Korea; zhangting92925@gmail.com (T.Z.); niyani0@naver.com (X.W.); yuanheng.changan@gmail.com (H.Y.); huangsk0606@gmail.com (S.H.); 2 Research Division of Food Functionality, Korean Food Research Institutes, Wanju 55365, Korea; kmj@kfri.re.kr (M.J.K.); yhj@kfri.re.kr (H.J.Y.); 3 Department of R&D, Unimed Pharmaceuticals Inc., Unimed Bldg., Seoul 05567, Korea; alswn0512@unimed.co.kr (M.J.K.); smyoon@unimed.co.kr (S.M.Y.); knkim@unimed.co.kr (K.-N.K.); 4 Department of Food and Nutrition, Obesity/Diabetes Research Center, Hoseo University, Asan 31499, Korea

**Keywords:** scopolamine, memory deficit, porcine brain enzyme hydrolysates, insulin resistance, acetylcholinesterase

## Abstract

No study has revealed the effect of porcine brain enzyme hydrolysate (PBEH) on memory impairment. We aimed to examine the hypothesis that PBEH intake modulates memory deficits and cognitive behavior in scopolamine (SC)-induced amnesia rats, and its mechanism, including gut microbiota changes, was determined. Sprague–Dawley male rats had intraperitoneal injections of SC (2 mg/kg body weight/day) at 30 min after daily feeding of casein (MD-control), PBEH (7 mg total nitrogen/mL) at 0.053 mL (Low-PBEH), 0.159 mL (Medium-PBEH), 0.478 mL (High-PBEH), or 10 mg donepezil (Positive-control) per kilogram body weight per day through a feeding needle for six weeks. The Normal-control rats had casein feeding without SC injection. PBEH dose-dependently protected against memory deficits determined by passive avoidance test, Y-maze, water-maze, and novel object recognition test in SC-induced rats compared to the MD-control. The High-PBEH group had a similar memory function to the Positive-control group. Systemic insulin resistance determined by HOMA-IR was lower in the PBEH groups than in the Normal-control but not the Positive-control. In parallel with systemic insulin resistance, decreased cholesterol and increased glycogen contents in the hippocampus in the Medium-PBEH and High-PBEH represented reduced brain insulin resistance. PBEH intake prevented the increment of serum TNF-α and IL-1β concentrations in the SC-injected rats. Hippocampal lipid peroxide and TNF-α contents and mRNA TNF-α and IL-1β expression were dose-dependently reduced in PBEH and Positive-control. PBEH decreased the hippocampal acetylcholinesterase activity compared to the MD-control, but not as much as the Positive-control. PBEH intake increased the α-diversity of the gut microbiota compared to the MD-control, and the gut microbiota community was separated from MD-control. In metagenome function analysis, PBEH increased the energy metabolism-related pathways of the gut microbiota, including citric acid cycle, oxidative phosphorylation, glycolysis, and amino acid metabolism, which were lower in the MD-control than the Normal-control. In conclusion, alleviated memory deficit by PBEH was associated potentially with not only reducing acetylcholinesterase activity but also improving brain insulin resistance and neuroinflammation potentially through modulating gut microbiota. PBEH intake (1.5–4.5 mL of 7 mg total nitrogen/mL for human equivalent) can be a potential therapeutic agent for improving memory impairment.

## 1. Introduction

The prevalence of dementia due to Alzheimer’s disease and cerebral stroke is rising as many countries become aging societies. Although the cause of the neurodegenerative disease is unknown, it is associated with the amyloid-β plaque and neurofibrillary tangles by tau hyperphosphorylation and neurotransmission disturbance [1]. Elevated brain insulin resistance, increased oxidative stress, and inflammation induces tau hyperphosphorylation, a microtubule-associated protein, to develop brain cell death and shrinkage. Among the neurotransmitters, cholinergic neurons are involved in attention, learning and memory functions, stress response, and sensory process [2]. The reduction of cholinergic transmitters, primarily acetylcholine, is related to memory dysfunction and cognitive impairment, progressing to Alzheimer’s disease [1,2]. In contrast, acetylcholinesterase inhibitors improve the symptoms of Alzheimer’s disease [3]. Stress is also related to the release of acetylcholine by modulating the hypothalamic–pituitary–adrenal (HPA) axis involved in the gut–brain axis [2]. After acetylcholine is released from the presynaptic neurons to the synaptic cleft, acetylcholine binds to the nicotinic or muscarinic receptors to provide biological function. The inhibition of acetylcholinesterase activity potentially improves learning and memory function because acetylcholinesterase hydrolyzes rapidly into acetate and choline in the synaptic cleft [2]. Therefore, acetylcholine synthesis and degradation regulate cholinergic neuron activation to modulate cognitive function.

Scopolamine is a non-selective post-synaptic muscarinic receptor blocker that occupies the muscarinic receptor with high affinity and activates acetylcholinesterase in the cortex and hippocampus [4]. It also decreases cerebral blood flow due to the cholinergic hypofunction and elevates oxidative stress and inflammation in the brain, particularly the hippocampus [5]. As a result, scopolamine induces neurotoxicity to disturb the learning and memory function, contributing to amnesia and Alzheimer’s-type dementia in animals and humans [6,7,8], even though its low dosage is used to suppress motion sickness in cars and ships. Therefore, an intraperitoneal scopolamine administration to a rat is a suitable animal model for assessing memory deficit and exploring the efficacy of potential therapeutic compounds.

Considerable research has been conducted to investigate the natural products and prevent or slow the progression of amnesia and Alzheimer’s-type dementia [9]. Most studies have demonstrated flavonoids, including luteolin, ellagic acid, ferulic acid, and L-theanine, as potential therapeutic compounds [10,11,12]. On the other hand, peptides from silk protein and porcine placenta and brain have been studied to demonstrate the improvement of learning and memory function with different pathways in animal and human studies [13,14,15,16]. Hence, porcine brain hydrolysates can improve learning ability and memory function. Furthermore, emerging evidence suggests a bidirectional interaction between brain function and gut microbiota [17]. Most studies showed that the probiotic and prebiotic intakes improve scopolamine-induced learning and memory disturbance by reducing the proinflammatory cytokines in animal models [18,19]. These studies reported that scopolamine-induced memory deficit improves memory impairment by inhibiting neuroinflammation and increasing neuronal cell survival but not by suppressing acetylcholinesterase activity, suggesting that probiotics and prebiotics indirectly alleviate memory dysfunction.

On the other hand, no study has examined the ability of porcine brain enzyme hydrolysate (PBEH) by protease in a scopolamine-induced animal model to inhibit the acetylcholine activity and modulate the gut–brain axis. This study hypothesized that the PBEH supplementation modulated memory deficit and abnormal cognitive behavior in the amnesia animal model and was associated with gut microbiota changes. The hypothesis was examined in scopolamine-induced amnesia rats, and the potential mechanism of PBEH was also revealed. This study reports a potential functional food that protects against memory impairment.

## 2. Results

### 2.1. Porcine Brain Enzyme Hydrolysate (PBEH) Amino Acid Composition

Total nitrogen contents of PBEH contained 6.95 ± 0.56 mg/mL, and its peptide contents (2–5 kd) were 65%. PBEH mainly contained L-arginine (2.36 ± 0.55 mg/mL), L-leucine (2.31 ± 0.42 mg/mL), and L-phenylalanine (1.61 ± 0.17 mg/mL) (Table 1).

### 2.2. Memory Deficit 

**Figure 1:**
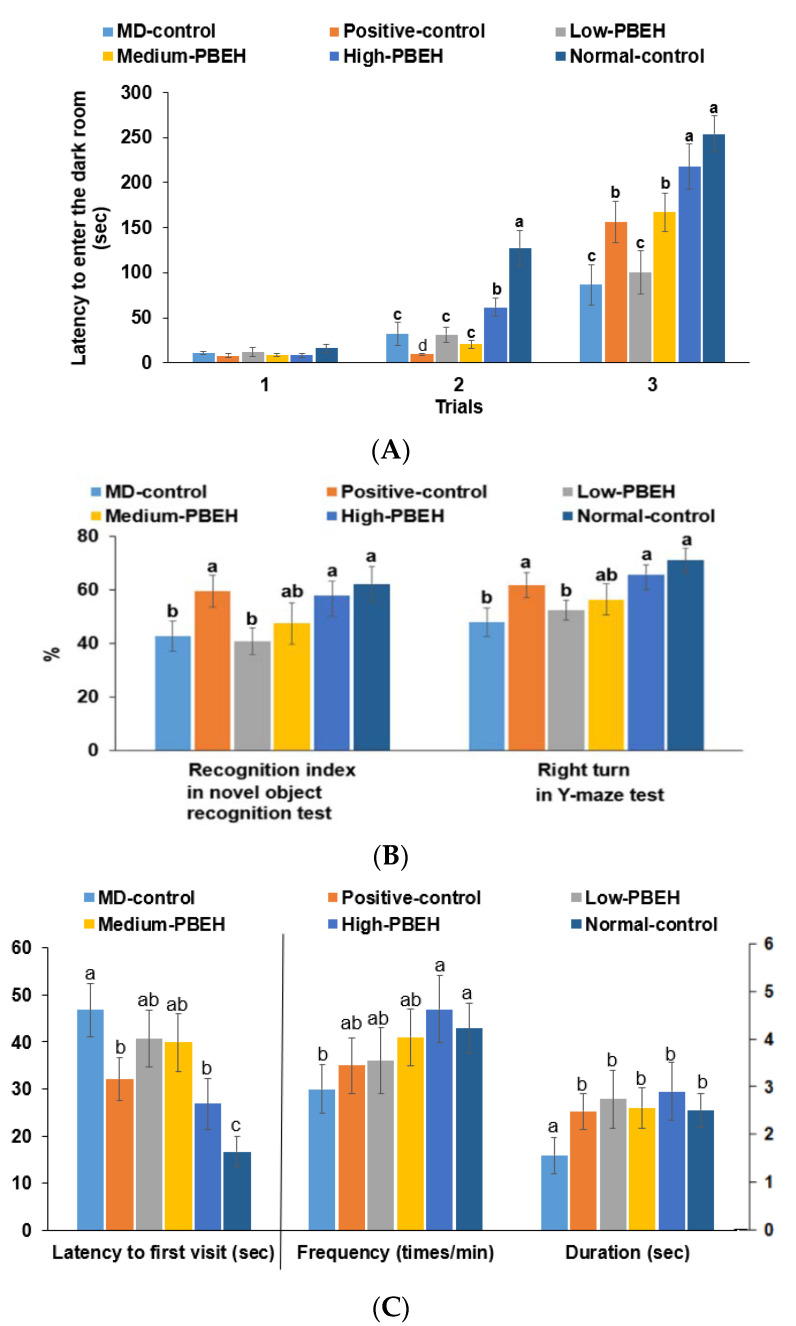
Memory deficits in rats after scopolamine injection. Male rats had intraperitoneal injections of scopolamine (2 mg/kg body weight/day) at 30 min after daily feeding of casein (MD-control), porcine brain enzyme hydrolysates (PBEH; 7 mg total nitrogen/mL) at 0.053 mL (Low-PBEH), 0.159 mL (Medium-PBEH), 0.478 mL (High-PBEH), or 10 mg donepezil (Positive-control) per kilogram body weight per day for six weeks. (**A**) Latency time to enter the dark box (s) during the first, second, and third trials; (**B**) novel object recognition index during novel objective recognition test and the number of the right turns among the total turns in the Y-maze test; (**C**) latency time to the first visit, frequencies to visit zone 5, and duration in zone 5. The bars and error bars represent the means ± standard deviations (*n* = 10). ^a–c^ Different superscript letters on the bars indicate a significant difference among the groups according to a Tukey test at *p* < 0.05.

The scopolamine intraperitoneal injection-induced memory deficit was measured by passive avoidance, Y-maze, novel object recognition tests for short-term memory, and water-maze test for spatial memory. In the second trial of the passive avoidance test, the Normal-control had a higher latency time than the control, and only the High-PBEH extended the time (Figure 1A). In the third trial, the latency time to enter the dark box was much lower in the MD-control than Normal-control, but it increased in the Positive-control, Medium-PBEH, and High-PBEH. The latency time in the High-PBEH was close to the Normal-control (Figure 1A). The novel object recognition test showed that the MD-control exhibited a lower novel object recognition rate than the Normal-control while the Positive-control and High-PBEH groups protected the decrease as much as the Normal-control (Figure 1B).

In the water-maze test, latency time to the first visit in zone 5, where the platform was placed, was longer in the MD-control than the Normal-control, while the donepezil and High-PBEH treatments reduced the latency time to find zone 5 compared to the Normal-control. The frequency and duration to visit zone 5 were lower in the MD-control than the Normal-control, whereas donepezil and PBEH supplementation prevented the decrease as much as the Normal-control (Figure 1C).

### 2.3. Depression

**Figure 2:**
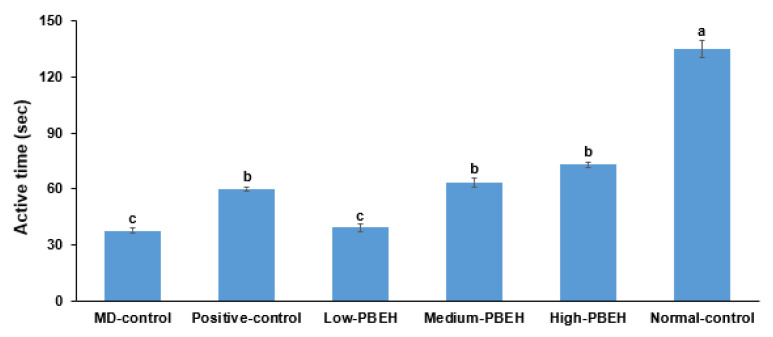
Depression measurement by forced swimming test. Male rats had intraperitoneal injections of scopolamine (2 mg/kg body weight/day) at 30 min after daily feeding of casein (MD-control), porcine brain enzyme hydrolysates (PBEH; 7 mg total nitrogen/mL) at 0.053 mL (Low-PBEH), 0.159 mL (Medium-PBEH), 0.478 mL (High-PBEH), or 10 mg donepezil (Positive-control) per kilogram body weight per day for six weeks. Bars and error bars represent the means ± standard deviations (*n* = 10). ^a–c^ Different superscript letters on the bars indicate a significant difference among the groups by Tukey test at *p* < 0.05.

Scopolamine-developed depression was indicated by decreasing retaining swimming rate, which was much lower in the MD-control than the Normal-control (Section 2.4). Positive-control (donepezil), Medium-PBEH, and High-PEBH treatments elevated the swimming rate, but it was not as high as the Normal-control (Figure 2).

### 2.4. Energy Metabolism and Glucose Metabolism

Memory deficit can be modified with systemic and brain insulin resistance, which may change energy and glucose metabolism. However, final body weight and weight gains during six weeks were not significantly different among the MD-control, Positive-control, and Normal-control groups, while they lowered in the Medium-PBEH and High-PBEH groups compared to the MD-control (*p* < 0.05; Table 2). Visceral fat mass adding epididymal fat and retroperitoneal fat masses showed a similar pattern of weight gain, and High-PBEH decreased visceral fat mass compared to the MD-control (*p* < 0.05; Table 2). The food efficiency was higher in the Positive-control group than the MD-control and Normal-control groups, while PBEH decreased food efficiency dose-dependently.

Serum glucose concentration at the fasting state was similar in the MD-control, Normal-control, and PBEH groups, but it increased in the Positive-control group (*p* < 0.05; Table 2). Fasting serum insulin concentration was much higher in the MD-control and PBEH groups than the Normal-control, while it lowered more in the donepezil group than the MD-control, but not as much as the Normal-control (*p* < 0.05; Table 2). The homeostatic model assessment for insulin resistance (HOMA-IR; insulin resistance index) was much higher in the MD-control than the Normal-control, and Low-PBEH, Medium-PBEH, and Positive-control did not differ from the MD-control (*p* < 0.05; Table 2). On the other hand, High-PBEH showed a lower HOMA-IR than the MD-control. Therefore, memory deficit elevated insulin resistance without changing body weight gain, and only High-PBEH decreased it but not as much as the Normal-control.

**Figure 3:**
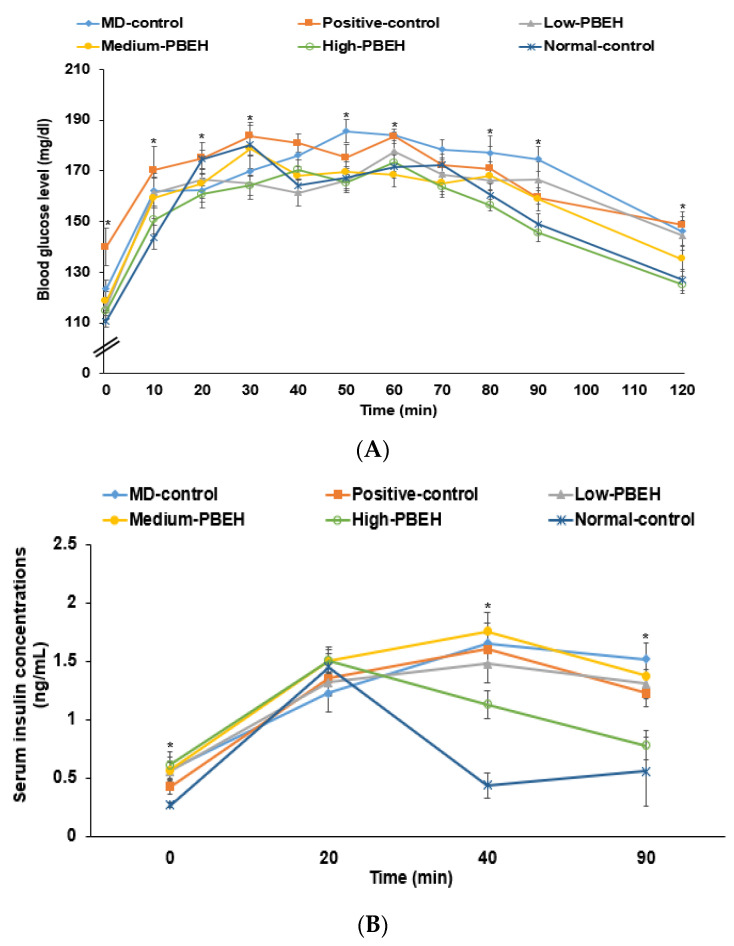
Changes in serum glucose and insulin concentrations and areas under the curve during oral glucose tolerance testing (OGTT) and intraperitoneal insulin tolerance testing (IPITT). Male rats had intraperitoneal injections of scopolamine (2 mg/kg body weight/day) at 30 min after daily feeding of casein (MD-control), porcine brain enzyme hydrolysates (PBEH; 7 mg total nitrogen/mL) at 0.053 mL (Low-PBEH), 0.159 mL (Medium-PBEH), 0.478 mL (High-PBEH), or 10 mg donepezil (Positive-control) per kilogram body weight per day for six weeks. (**A**) Changes in serum glucose concentration during oral glucose tolerance test after oral consumption of 2 g glucose/kg body weight; (**B**) changes of serum insulin concentrations during OGTT; dots and error bars represent the means ± standard deviations (*n* = 10). * Significantly different among the groups at each time point at *p* < 0.05.

After glucose loading in the oral glucose tolerance test (OGTT), the serum glucose concentrations increased until 30–40 min, and they decreased in all rats (Figure 3A). The serum glucose concentrations increased until 50 min and decreased slowly in the MD-control. The serum glucose concentrations increased until 30 min in the Positive-control group while they decreased until 90 min but not after 90 min. PBEH decreased the serum glucose concentrations after 40 min, and High-PBEH lowered them as much as the Normal-control (Figure 3A). These results indicated that rats in the MD-control showed glucose intolerance and High-PBEH normalized glucose tolerance similar to the Normal-control. The serum insulin concentrations in the Normal-control group were elevated at 30 min, then decreased rapidly until 40 min, and were relatively constant after that. However, in the MD-control and Positive-control groups, they increased until 40 min and decreased slowly until 90 min (Figure 3B). A High-PEBH improved serum insulin concentrations, as much as the Normal-control.

### 2.5. Proinflammatory Cytokines and Liver Damage Index in the Circulation

Memory deficit is also associated with neuroinflammation interacted with systemic inflammation. The serum tumor necrosis factor-α (TNF-α) concentrations were higher in the MD-control than in the Normal-control, but they decreased in the Medium-PBEH, High-PBEH, and Positive-control groups (Table 3). The serum interleukin-1β (IL-1β) concentrations showed similar patterns of serum TNF-α concentrations, but they did not decrease in the Positive-control (Table 3). These results indicated that Medium-PBEH and High-PBEH reduced systemic inflammation as much as the Normal-control. The serum alanine aminotransferase (ALT) and aspartate aminotransferase (AST) concentrations were similar in the MD-control and Normal-control, and they were not altered in the Positive-control, Low-PBEH, and Medium-PBEH groups. On the other hand, High-PBEH rather lowered their concentrations (Table 3). PBEH and donepezil did not show any signs of liver damage.

### 2.6. Hippocampal Lipids, Oxidative Stress, and Neuroinflammation by Scopolamine Injection

The hippocampal insulin resistance, oxidative stress, and neuroinflammation are associated with memory deficits. Brain triglyceride, cholesterol, and glycogen contents indirectly represent brain insulin resistance. The hippocampal triglyceride contents were not significantly different among the groups but tended to be lower in the Medium-PEBH than in the MD-control (Table 4). On the other hand, MD-control had higher hippocampal cholesterol contents than the Normal-control. Positive-control, Medium-PBEH, and High-PBEH groups lowered them, but not as low as the Normal-control (Table 4). Furthermore, hippocampal glycogen contents were lower in the MD-control than Normal-control, while donepezil and PBEH increased the contents as much as the Normal-control (Table 4). Thus, brain insulin resistance was higher in the MD-control than Normal-control while Medium-PBEH and High-PBEH decreased it.

Hippocampal lipid peroxide contents, indicating brain oxidative stress, were higher in the MD-control than the Normal-control, and donepezil and PBEH lowered the contents. The High-PBEH group showed a decrease to as low as the Normal-control (Table 4). The acetylcholinesterase activity to remove acetylcholine was higher in the MD-control than the Normal-control after the scopolamine injection, and donepezil reduced it the most, but not as much as the Normal-control. The Medium-PBEH and High-PBEH groups also showed a lower activity, but they did not decrease it as low as the Positive-control (Table 4).

The TNF-α contents in the hippocampus lysate showed a similar pattern to the lipid peroxide contents, but their contents in the PBEH did not reach those in the Normal-control (Table 4). In addition, hippocampal mRNA expression of *TNF-α* and *IL-1β* was higher in the MD-control than the Normal-control, and High-PBEH lowered them as much as the Normal-control (Table 4). Donepezil decreased the hippocampal mRNA expression of *TNF-α*, but not *IL-1β*. The relative mRNA expression of hippocampal brain-derived neurotrophic factor (*BDNF*) was lower in the MD-control than the Normal-control, while Positive-control and High-PBEH increased it to a similar level to the Normal-control (Table 4). Ciliary neurotrophic factor (*CNTF*) mRNA expression showed a similar pattern to *BDNF*, but its expression was lower in the MD-control than the Normal-control. The increase by PBEH and donepezil did not reach the Normal-control (Table 4). Therefore, neuroinflammation was elevated in the MD-control compared to the Normal-control, and it was normalized with Medium-PBEH and High-PBEH.

### 2.7. Gut Microbiota and Short-Chain Fatty Acids (SCFA)

**Figure 4:**
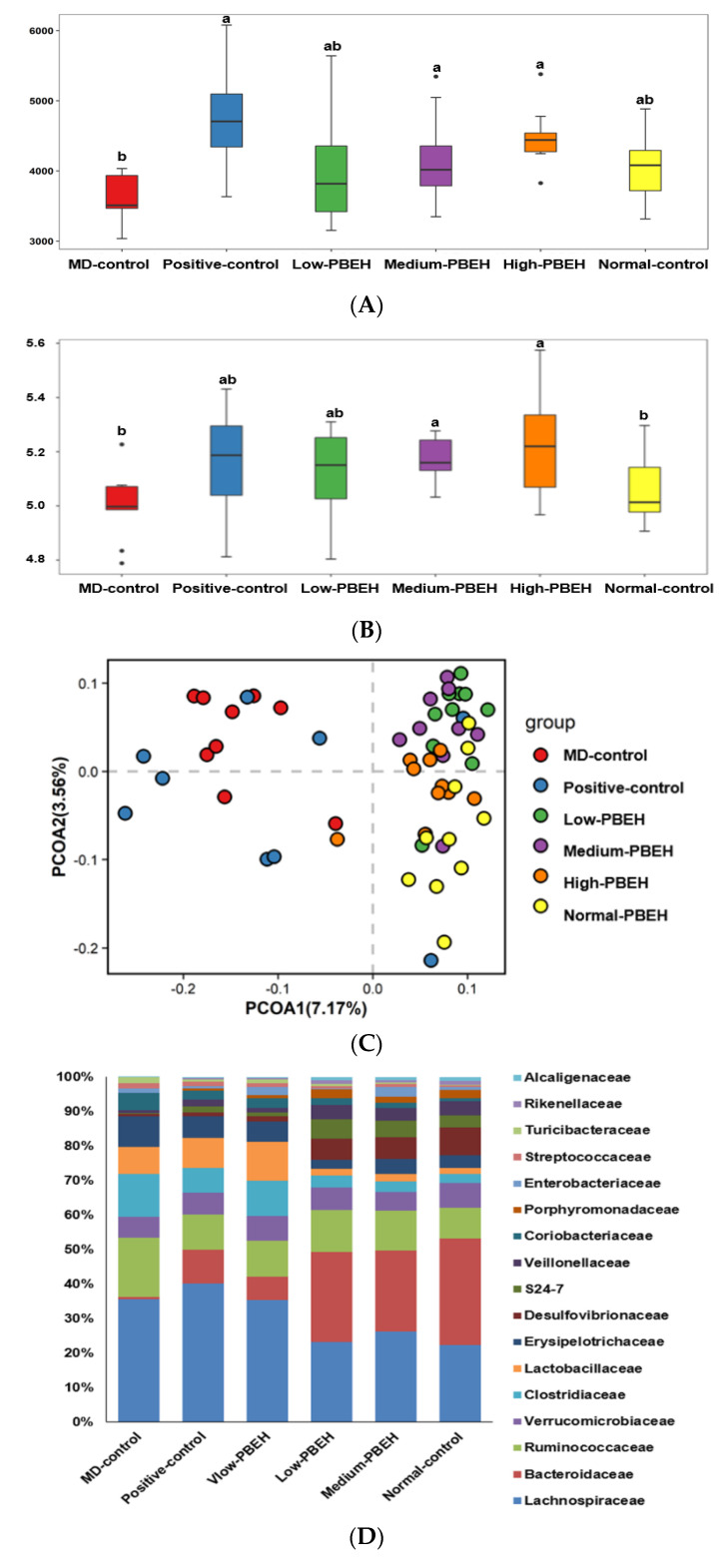

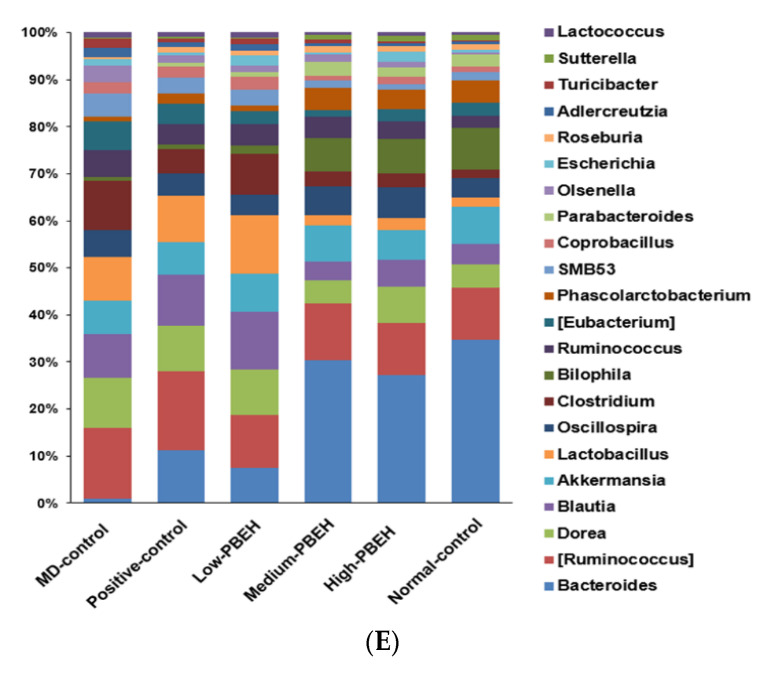
Gut microbiota α- and β-diversity and profiles. Male rats had intraperitoneal injections of scopolamine (2 mg/kg body weight/day) at 30 min after daily feeding of casein (MD-control), porcine brain enzyme hydrolysates (PBEH; 7 mg total nitrogen/mL) at 0.053 mL (Low-PBEH), 0.159 mL (Medium-PBEH), 0.478 mL (High-PBEH), or 10 mg donepezil (Positive-control) per kilogram body weight per day for six weeks. (**A**) Chao1 index of fecal bacteria; (**B**) Shannon index of fecal bacteria; (**C**) principal coordinate analysis (PCoA) of fecal bacteria; (**D**) relative amounts (%) of fecal samples at the family level; (**E**) relative amounts (%) of fecal samples at the genus level. Bars and error bars represent the means ± standard deviations (*n* = 10). ^a,b^ Different superscript letters on the bars indicated a significant difference among the groups by Tukey test at *p* < 0.05.

The systemic and brain insulin resistance and inflammation are well known to be related to the gut–brain axis. The Chao1 and Shannon indices were used to determine the α-diversity, indicating gut microbiota richness. Both were lower in the MD-control than the Normal-control and higher in the Positive-group than the MD-control. PBEH also increased both indices, and the increment was higher in the Medium- and High-PBEH than the Normal-control (Figure 4A,B). The clustering of the gut bacterial community by principal coordinate analysis (PCoA) is helpful to show the effect of PBEH on fecal bacteria because the gut microbiota is large and complex (Figure 4C). The bacterial clustering of the MD-control group showed significant separation from the Normal-control, Positive-control, and PBEH groups. The fecal bacteria were well separated in the MD-control and Normal-control, while the PBEH and the Normal-control groups were closely located compared to the MD-control (Figure 4C).

The bacteria community at the family and genus levels showed different patterns between the MD-control and Normal-control groups. PBEH intake changed the bacteria composition toward the Normal-control (Figure 4D). *Bacteroides* were much lower in the MD-control than the Normal-control, and the Positive-control and PEBH intake increased *Bacteroides* at the genus level (Figure 4E). However, MD-control increased *Ruminococous* and *Dorea* in the family Lachnospiraceae compared to the Normal-control, and they were lower in the Medium- and High-PBEH groups (Figure 4D,E). On the other hand, *Lactobacillus* was higher in the MD-control than the Normal-control and lowered in the Medium- and High-PBEH (Figure 4E). Thus, the gut bacteria community was different in the MD-control and Normal-control. PBEH changed it similar to the Normal-control, but it was difficult to determine which gut microbiota community was better among the groups.

**Figure 5:**
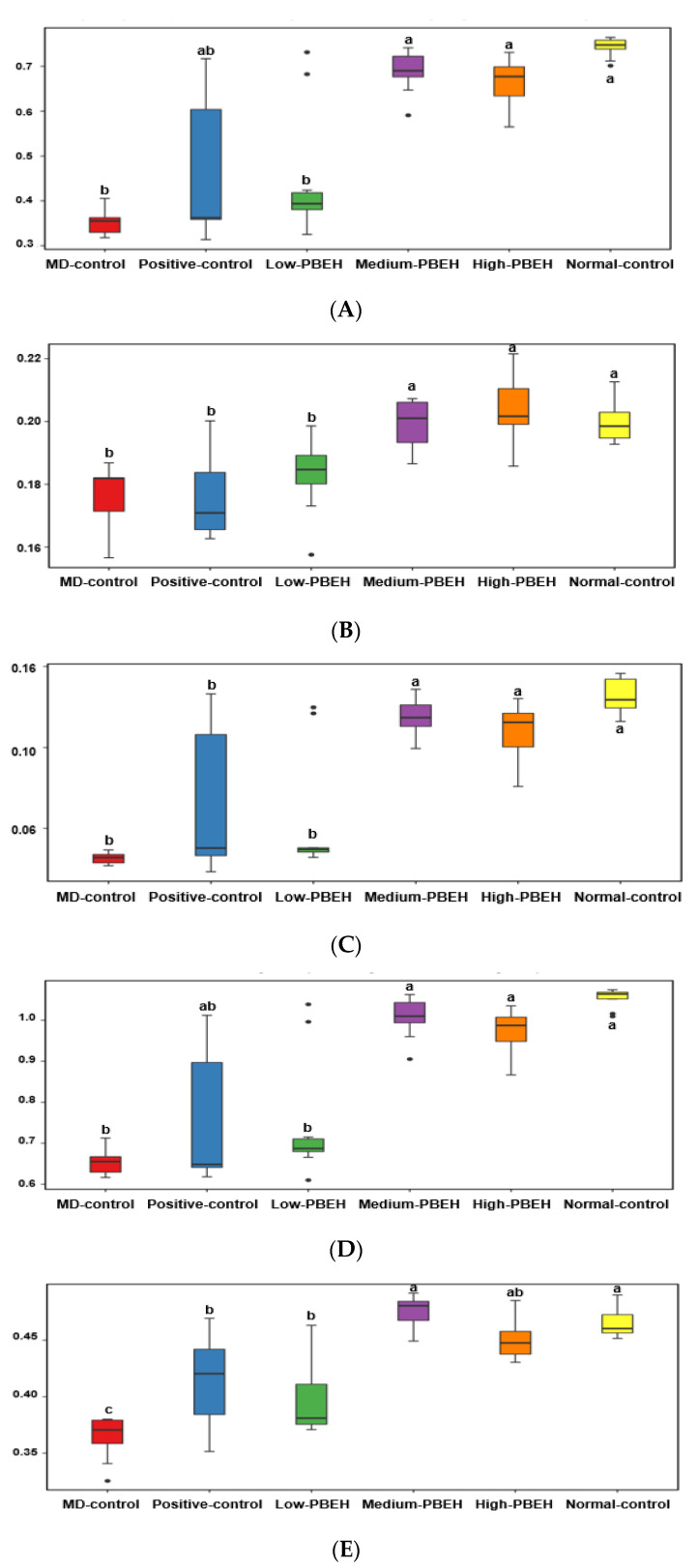

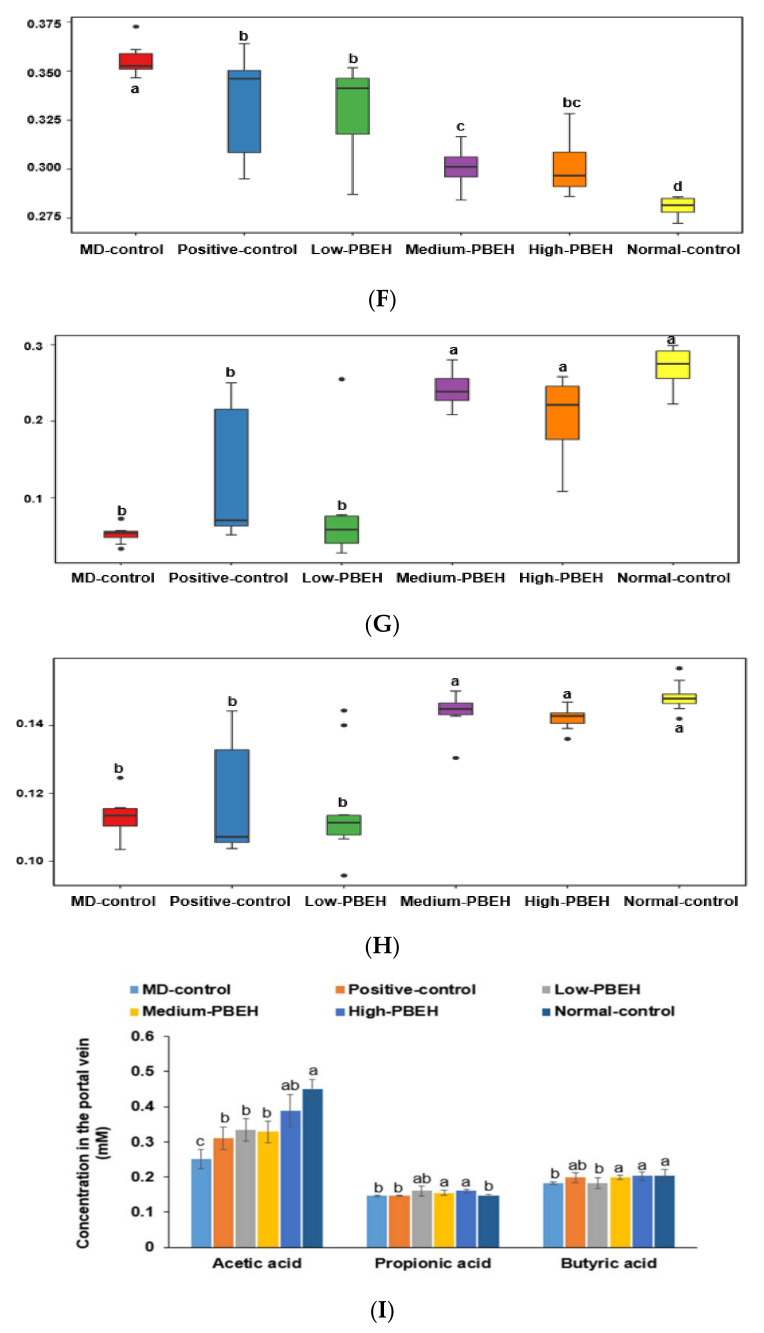
Gut microbiome functions and short-chain fatty acids in the portal vein. Male rats had intraperitoneal injections of scopolamine (2 mg/kg body weight/day) at 30 min after daily feeding of casein (MD-control), porcine brain enzyme hydrolysates (PBEH; 7 mg total nitrogen/mL) at 0.053 mL (Low-PBEH), 0.159 mL (Medium-PBEH), 0.478 mL (High-PBEH), or 10 mg donepezil (Positive-control) per kilogram body weight per day for six weeks. (**A**) Glycolysis (Embden–Meyerhof pathway), glucose → pyruvate; (**B**) pyruvate oxidation, pyruvate→ acetyl-CoA; (**C**) beta-oxidation, acetyl-Co A synthesis; (**D**) citrate cycle (TCA cycle); (**E**) leucine degradation, leucine → acetoacetate + acetyl-CoA; (**F**) fatty acid biosynthesis, initiation; (**G**) bile acid biosynthesis, cholesterol → cholate/chenodeoxycholate; (**H**) 3-Hydroxypropionate bi-cycle. (**I**) Bars and error bars represent the means ± standard deviations (*n* = 10). ^a–d^ Different superscript letters on the bars indicate a significant difference among the groups by Tukey test at *p* < 0.05.

The metagenome function was determined with the Picrust2 program. Interestingly, the energy metabolism, including glycolysis, β-oxidation, and citrate cycle, was lower in the MD-control than the Normal-control and PBEH prevented the decrease in energy metabolism (Figure 5A–H). The degradation of some amino acids, including leucine, to generate acetyl CoA was higher in the Medium-PBEH and High-PBEH than the MD-control, while it was lower in the MD-control than the Normal-control. On the other hand, fatty acid synthesis and bile acid biosynthesis were higher in the MD-control than the Normal-control and lowered in the Medium-PBEH and High-PBEH. These results show that MD-control decreased energy and cholesterol utilization and increased fatty acid biosynthesis, and the Medium-PBEH and High-PBEH prevented the changes by the scopolamine injection. The acetic acid contents in the portal vein blood were much lower in the MD-control than in the Normal-control, and PEBH administration elevated them dose-dependently (Figure 5I). Propionate and butyrate concentrations were similar in the MD-control and Normal-control but higher in the Medium- and High-PBEH groups than the MD-control (Figure 5I).

## 3. Discussion

The present study showed that PBEH supplementation improved memory deficit and cognitive behavior in scopolamine-induced amnesia rats. Although PBEH acted as the moderate acetylcholinesterase inhibitor, it reduced brain insulin resistance and neuroinflammation to protect against memory impairment by scopolamine. High-PBEH showed similar activity to donepezil, a potent suppressor of acetylcholinesterase activity. The scopolamine injection also changed the gut microbiota to decrease the α-diversity, and PBEH protected against the modification of the gut microbiota in the scopolamine-injected rats. In metagenome analysis, PBEH intake increased gut microbiota involved in energy metabolism, including the tricarboxylic acid cycle (TCA), glycolysis, oxidative phosphorylation, and some amino acid degradation compared to the MD-control. It might be related to lower weight gain in PBEH groups than MD-control. The present study suggested that PEBH could be a potential functional food to protect against memory impairment.

Scopolamine is a non-selective post-synaptic muscarinic receptor blocker to activate acetylcholinesterase to impair cholinergic neurotransmission [20]. The modulation of acetylcholinesterase activity plays a crucial role in memory impairment. As a result, an acute scopolamine injection induces transient memory dysfunction such as forgetfulness, which is the early sign of Alzheimer’s disease. Its long-term injection progresses to memory impairment and behavior disturbance to cause Alzheimer’s disease-like dementia [21]. Thus, the animal model in the present study progressed from the acute stage of memory deficit to Alzheimer’s disease-like symptoms. Scopolamine is also reported to increase oxidative stress in the brain, and bioactive components to reduce oxidative stress, such as flavonoids (apigenin and silymarin), prevent memory impairment by scopolamine [22,23]. Their intake reduces reactive oxygen species and increases glutathione (GSH), similar to donepezil. Porcine brain extract intake has been demonstrated to improve brain functions, including the neurological score, motor performance, and memory deficit in cerebral ischemic rats with middle cerebral artery occlusion by decreasing lipid peroxides with increasing antioxidant enzymes, including superoxide dismutase, catalase, and GSH peroxides [24]. Porcine brain hydrolysate intake also ameliorates the memory deficit in amyloid-β (1–40) infused rats to induce Alzheimer’s disease-like dementia by suppressing acetylcholinesterase activity, lipid peroxides, and amyloid-β accumulation [15]. However, there was no study of PBEH in scopolamine-induced memory impairment. PBEH reduced lipid peroxide contents and *BDNF* expression in the hippocampus by inhibiting the acetylcholinesterase activity and reducing proinflammatory cytokines in the present study. These changes by PBEH were associated with reversing the memory impairment by scopolamine injection.

Donepezil is a potent acetylcholinesterase inhibitor, and the US FDA approved donepezil for dementia treatment, but its long-term efficacy and safety are unclear. Diet interventions such as protein hydrolysates have been studied for dementia prevention [25]. In the present study, donepezil was used as the Positive-control. PBEH acted as a suppressor of acetylcholinesterase, but its efficacy was lower than donepezil. PBEH protected against scopolamine-induced memory impairment dose-dependently, and the high-PBEH improved it as much as donepezil. Nevertheless, the acetylcholinesterase activity was lower in the Positive-control (donepezil) than High-PBEH. These results in the present study indicated improved memory function by PBEH was associated with not only inhibition of the acetylcholinesterase activity but also other factors related to IL-1β, oxidative stress, and insulin resistance in the brain.

Alzheimer’s disease is associated with decreased glucose utilization in the brain related to insulin resistance [26]. Donepezil disturbed the glucose metabolism by reducing insulin secretion at the acute phase during OGTT, while PBEH improved the glucose metabolism in the present study. It might be associated with increasing the acetylcholine level in the peripheral tissues by donepezil treatment, influencing glucose metabolism. Acetylcholine stimulates the insulin-secreting β-cell via the muscarinic acetylcholine receptors, M3 and M5, and the somatostatin-secreting δ-cell via M1 receptors to suppress insulin secretion. Thus, the increase in acetylcholine by inhibiting acetylcholinesterase activity may inhibit the somatostatin-secreting δ-cell via M1 receptors, which is associated with inhibiting insulin secretion. Lin et al. reported that donepezil and metformin administration improves cognitive function and insulin resistance but not donepezil plus acarbose in patients with cognitive impairment and abnormal glucose metabolism [27]. These results suggest that donepezil does not improve insulin resistance. However, since PBEH acted as a moderate acetylcholinesterase inhibitor, it did not suppress insulin secretion and decreased insulin resistance. Therefore, PBEH improved cognitive function by suppressing acetylcholinesterase activity and promoting brain insulin sensitivity in rats with a scopolamine-induced memory deficit.

Neurodegenerative diseases can be prevented by changing the daily diet to modulate the microbiota–gut–brain axis [10]. The gut–brain axis involves two-way biochemical signaling between the gastrointestinal tract and the central nervous system (CNS). The gut microorganisms contribute to the host’s energy metabolism, serum metabolites, and brain function through SCFA, secondary bile acids, amino acids, and neurotransmitters [25,28]. The dysbiosis of the gut microbiota decreasing *Lactobacillus* and *Bifidobacterium* species is related to neurodegenerative diseases [29]. PBEH increased the α-diversity, and the gut microbiota in the PBEH groups was separated from the MD-control in the present study. Gut microbiota in the Medium- and High-PBEH were changed into that of the Normal-control: *Lactobacillus*, *Blautia*, and *Dorea* decreased, and *Biophilia* and *Bacteriodes* increased in the Medium- and High-PBEH as much as the Normal-control.

Interestingly, their changes caused a significant alteration in metagenomic function determined by Picrust2. The gut microbiome indicated that energy metabolism-related pathways differed in the MD-control and Normal-control. However, MD-control and Normal-control had similar body weight, and it indicated that scopolamine altered energy metabolism and gut microbiota changes compensated it. By contrast, PBEH intake in MD rats decreased the body weight and visceral fat mass compared to the MD-control without decreasing the food intake. These results suggest that energy expenditure might be elevated in PBEH, but it is not applied to MD-control and Normal-control. The metagenome data suggested that the Medium- and High-PBEH intake elevated glycolysis, citric acid cycle, β-oxidation, and some amino acid degradation compared to the MD-control. The increment in the PBEH groups was similar to the Normal-control. Therefore, the body weight changes might be related directly to the gut microbiota changes in a scopolamine injection, and PBEH intake reversed its changes. In addition, the gut–brain axis influences memory function and weight regulation [30].

In conclusion, a long-term scopolamine injection induced memory dysfunction, and PBEH intake (10–30 mg/day for human equivalent) protected against its induction as much as the donepezil treatment. Although PBEH and donepezil inhibited acetylcholinesterase activity in the brain, PBEH was a weaker inhibitor than donepezil. However, PBEH improved insulin resistance and reduced the lipid peroxide and IL-1β contents in the hippocampus better than donepezil. PBEH and donepezil did not show adverse effects, including liver damage. Therefore, with additional clinical research, PBEH can be an alternative therapy for alleviating human memory deficits. 

## 4. Materials and Methods

### 4.1. Porcine Brain Peptides

Blood was removed from a porcine brain with distilled water, and ethanol was added to dissolve fat components and precipitate proteins. The precipitates were digested with protease at 37 °C for 26 h, which was inactivated. Undigested proteins were precipitated with ethanol and filtered using the ultrafiltration method. The filtrates showed a 58% yield. The filtrates (PBEH) mainly contained 2–5 kd peptides, and they were used for the animal feeding study. As described previously, amino acid contents in PBEH were determined using an amino acid analyzer (L-8500; Hitachi, Tokyo) [31]. Peptide contents were calculated using the equation: (total nitrogen content − total amino acids)/total nitrogen contents.

### 4.2. Animal Care and Animal Model for Scopolamine-Induced Memory Deficit

Seventy male Sprague–Dawley rats aged eight weeks (216 ± 13.0 g) were purchased from Daehan Bio Inc. (Eum-Sung, Korea). The rats were acclimatized for seven days in an animal facility. Each animal was housed in an individual stainless-steel cage in a controlled environment (23 °C, 12 h light/dark cycle) with access to food and water *ad libitum*. All procedures conformed with the Guide for the Care and Use of Laboratory Animals (8th edition) issued by the National Institutes of Health and were approved by the Institutional Animal Care and Use Committee of Hoseo University (HSIACUC-17071).

**Figure 6:**
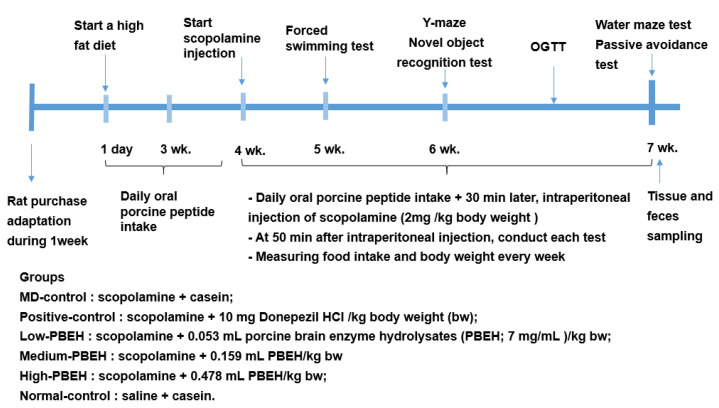
Experimental design.

Experimental design is presented in Figure 6. Rats were induced memory deficit by daily intraperitoneal (IP) injection of scopolamine solution. Scopolamine hydrobromide and donepezil hydrochloride (Sigma-Aldrich, St. Louis, MO, USA) were dissolved in 0.9% saline and distilled water, respectively, for administration into the experimental animals. At 30 min after the oral administration of casein (MD-control), porcine brain enzyme hydrolysates (PBEH; 7 mg total nitrogen/mL) at 0.053 mL (Low-PBEH), 0.159 mL (Medium-PBEH), or 0.478 mL (High-PBEH) per kg body weight by feeding needle, and scopolamine solution was injected IP to rats at 2 mg/kg body weight daily during the experimental periods. Donepezil (Positive-control) was administered orally at 10 mg/kg bw (equivalent to 10 mg/day human dose) into the rats through a feeding needle. At 30 min after the oral consumption of donepezil, the Positive-control and control rats had an IP injection of scopolamine to induce a memory deficit, while Normal-control rats were IP injected with saline. In the Normal-control groups, casein was provided orally to the rats, and saline was injected instead of scopolamine. Casein was made as 44 mg/mL and provided 0.478 mL for MD-control and Normal-control groups.

### 4.3. Diet Preparation

All rats had free access to a high-fat diet prepared using a modified polyphenol-free AIN-93 semi-purified method, which has been shown to induce insulin resistance and exacerbate memory deficits from scopolamine administration [32]. The semi-purified diets contained 37% carbohydrate (cornstarch and sucrose), 20% protein (casein), and 43% lard (CJ Co., Seoul, Korea), and vitamins and minerals were added. The feed was then re-sieved and stored at 4 °C. Each diet had equivalent energy (5.1 kcal/g) and nutrient composition. All rats had access to water and the experimental diets *ad libitum* during the entire experimental period.

### 4.4. Memory Deficit Assessments Using the Passive Avoidance, Y-Mase, and Water-Maze

A rat quickly entered the dark box in the passive avoidance apparatus equipped with a two-compartment dark/light shuttle box when left in the light shuttle box [33]. Electrostimulation (75 V, 0.2 mA, 50 Hz) was delivered to the rat when it entered the dark box in two acquisition trials by eight hours. After 16 h after the second trial, the latency time to enter the dark chamber was reassessed in the same manner, but no electric stimulation was delivered to the foot. Latency was measured up to a maximum of 600 s. The longer the latency time, the better the memory function.

The Y-maze test was conducted in a horizontal Y-shaped maze with three arms, 50.5 cm in length, 20 cm in width, and 20 cm in height. A rat was located in one arm, and its movements were monitored for 8 min. Consecutive entry into all three arms of the Y-maze was considered a correct alternation [34]. The percentage of spontaneous corrected alternations was calculated by dividing the correct alternations by the percentage of total arm entries.

The spatial memory function was evaluated using the Morris water-maze test [32,33], which assessed hippocampal-dependent learning, including the acquisition of spatial memory. At the start, a rat was located in zone 1 of the pool, and it tried to find the platform placed in zone 5. A water-maze test was conducted three times: a rat learned to find the platform located in zone 5 on days 1 and 2. On day 5, the latency time, frequency to visit, and duration in zone 5 were measured to evaluate spatial memory. The test was performed with a cut-off time of 600 s. 

### 4.5. Memory Deficit Assessments Using Novel Object Recognition Test 

All rats were subjected to a novel object recognition test for four days. A novel object recognition test was performed according to a previous research method with slight modifications [33,34]. Briefly, in the first three days of the experimental test, each rat was allowed to explore the field of the plexiglass box for 5 min (one day), 20 min (two days), and 20 min (three days) to adapt to the environment. During the last day of testing, the box was divided into four areas in the training phase, and two identical objects were placed in the center of the diagonal area. The rat was placed on the side of the box and allowed to explore objects freely for 10 min. After each test, the objects and instruments were cleaned with 75% ethanol to remove the rat odor. After one hour, a new object was used to replace one of the two identical objects in training, and the new object was similar in size but different in shape and color. The time spent by the rat exploring the new object and the familiar old object was recorded, respectively, within 5 min. The exploratory behavior was defined as a rat using its nose or front paws to smell and touch an object, but the rat sitting on the object or turning around was not considered exploration behavior. The result was expressed as the recognition index, which was the time exploring a new object divided by the total time to explore the old and new two objects. The recognition index (50%) was considered incidental. The higher recognition index indicated preferable object recognition memory.

### 4.6. Forced Swimming Test

Each rat had a 10 min pretest in a clear acrylic cylinder (60 cm in height and 30 cm in width) filled with up to 45 cm water at 24 ± 1 °C. After 24 h of the pretest, the 5 min swim test was performed to make a score for active (swimming and climbing) or passive (immobility) behavior when the rats were forced to swim in the cylinder. The researcher randomly and blindly selected and evaluated the video of the 5 min session and measured the time for total mobile and immobile behavior. The results were shown as the active time during the 5 min session.

### 4.7. OGTT

After an overnight fast, oral glucose administration (2 g/kg body weight) was conducted. The serum glucose concentrations were measured every 10 min for 90 min and again at 120 min using a Glucometer (Accuchek, Roche Diagnostics; Basel, Switzerland). The serum insulin concentrations in the fasting state were determined using an ultrasensitive enzyme-linked immunosorbent assay (ELISA) kit (Crystal Chem, Elk Grove Village, IL, USA). HOMA-IR was calculated as follows: serum insulin (μU) × serum glucose (mmol/L)/22.5.

### 4.8. Tissue Collection and Assays at the End of the Intervention

The next day after the water-maze test, all rats were deprived of food for 16 h to make metabolic parameters of the experimental animals not affected by previous food intake. Blood was drawn from the portal vein, the inferior vena cava, and organs were collected rapidly after anesthetizing with the mixture of ketamine and xylazine (100 and 10 mg/kg body weight). The epididymal and retroperitoneal fat masses were weighed. Six brains selected randomly from each group were frozen at −70 °C, while the rest (*n* = 4) were fixed in the 4% paraformaldehyde. The feces in the cecum were collected and frozen at −70 °C. 

The hippocampi were divided into two sections: one part was lysed with RIPA buffer containing protease inhibitors, and the other was randomly selected to be lysed with Trizol reagent (Invitrogen, Rockville, MD, USA) for extracting the total RNA. The hippocampal RIPA buffer lysates were centrifuged at 5000× *g* for 10 min, and their supernatants were used to measure the lipid peroxide and cholesterol contents by colorimetry using a lipid peroxidation (MDA) assay kit (Abcam, Cambridge, UK) and cholesterol kit (Asan Pharmaceutics). The supernatants were deproteinized with 1.5N perchloric acid, and the glycogen content was calculated from the glucose concentrations derived from glycogen hydrolyzed by α-amyloglucosidase in an acid buffer [35]. The glucose concentrations were measured using a glucose kit (Asan Pharmaceutics, Seoul, Korea). Triglyceride was extracted with a chloroform–methanol solution (2:1, v/v) from the hippocampus and resuspended in pure chloroform [36]. Triglyceride contents in the chloroform were measured using a triglyceride colorimetric kit (Asan Pharmaceutics, Seoul, Korea). Serum TNF-α and IL-1β concentrations were measured using an ELISA kit (eBioscience; San Diego, CA, USA), and serum concentrations of AST and ALT were assayed using the AST and ALT kits (Asan Pharmaceutics, Seoul, Korea), respectively.

### 4.9. Quantitative Real-Time PCR

cDNA was produced using a mixture of isolated total RNA from the hippocampus with superscript III reverse transcriptase and high-fidelity Taq DNA polymerase (1:1:1, *v:v:v*) using polymerase chain reaction (PCR). The cDNA was mixed with the primers for the genes of interest and SYBR Green mix to determine the expressions of the designated genes using a real-time PCR machine (Bio-Rad Laboratories, Hercules, CA, USA). As previously published, the primers used for the *CNTF*, *BDNF*, *TNF-α*, *IL-1β*, and *β-actin* were given [37]. The gene expression levels were quantitated using the comparative cycle of threshold (CT) method [38].

### 4.10. Serum SCFA Concentrations and Gut Microbiome by Next-Generation Sequencing (NGS)

The serum separated from the portal vein blood was mixed with ethanol (Duksan, Korea), and 1N HCl (100:1) was added to the mixture. The mixture was vortexed and centrifuged at 15,000 rpm, 15 min, and 4 °C. The SCFA concentrations in supernatants were measured by gas chromatography (GC, Clarus 680 GAS, PerkinElmer, Waltham, MA, USA) using an Elite-FFAP 30 m × 0.25 mm × 0.25 μm capillary column with helium as the carrier gas at a flow rate of 1 mL/min, as described previously [39]. Exogenous acetate, propionate, and butyrate (Sigma Co., St. Loise, MO, USA) were used as external standards.

The fecal microbiome communities were investigated using the feces from ceca by metagenome sequencing using next-generation sequencing procedures [40]. According to the manufacturer’s instructions, the bacterial DNA was extracted from the feces using a Power Water DNA Isolation Kit (Qiagen, Valencia, CA, USA). The DNA was amplified with the 16S amplicon primers by PCR, and libraries were prepared for PCR products according to the GS FLX plus library prep guide, as described previously [41]. According to the manufacturer’s instructions, the PCR amplification program was run with 16S universal primers in the FastStart High Fidelity PCR System (Roche, Basel, Switzerland). The sequencing of bacterial DNA in the feces was performed using the Illumina MiSeq standard operating procedure and a Genome Sequencer FLX plus (454 Life Sciences) (Macrogen, Seoul, Korea).

The 16S amplicon sequences were processed using Mothur v.1.36. MiSeq SOP was used to identify fecal bacterial taxonomy, and bacterial counts were conducted on each fecal sample. The sequences were aligned using Silva reference alignment v. 12,350, and bacteria counts and identifications for all taxa were determined, as described previously [39,41]. The relative bacteria counts were calculated in taxonomic assignment order for each sample. The PCoA results for gut bacteria were visualized using the R package.

### 4.11. Metabolic Functions by Gut Microbiome by PICRUSt2 Pipeline Analysis

The metabolic functions of the gut microbiota were predicted from the fasta files and count tables of fecal bacteria using PICRUSt2 [22]. The metabolic functions were based on the Kyoto Encyclopedia of Genes and Genomes (KEGG) Orthologues (KO) mapped using KEGG mapper (https://www.genome.jp/kegg/tool/map_pathway1.html, accessed on 11 November 2021) [41]. The gut microbiome was used to explore the differences in metabolic functions among the groups.

### 4.12. Statistical Analyses

The statistical analysis was performed using SAS version 7 (SAS Institute; Cary, NC, USA). A sample size of 10 per group was determined using the G power program (power = 0.85 and effect size = 0.50) to test the main effects. The results are expressed as the means ± standard deviations (SDs). Univariate analysis was used to check the normal distribution of variables, and one-way analysis of variance (ANOVA) was used to compare the significance of the groups when the variables were normally distributed. Multiple comparisons were conducted using a Tukey test when one-way ANOVA showed a significant intergroup difference. Statistical significance was accepted for *p* < 0.05.

## Figures and Tables

**Table 1:** Amino acid composition in porcine brain enzyme hydrolysates (PBEH).

Amino Acids	PBEH (mg/mL)	Amino Acids	PBEH (mg/mL)
ASP	0.487 ± 0.020	PRO	0.4835 ± 0.028
SER	0.590 ± 0.019	VAL	0.778 ± 0.033
GLU	0.686 ± 0.024	MET	0.790 ± 0.035
GLY	0.186 ± 0.020	LYS	2.170 ± 0.085
HIS	0.445 ± 0.029	ILU	0.868 ± 0.022
ARG	2.360 ± 0.115	LUE	2.308 ± 0.0628
TRE	0.348 ± 0.004	PHE	1.609 ± 0.025
ALA	0.664 ± 0.035		

**Table 2:** Body weight and glucose metabolism at the end of experimental periods.

	MD-Control	Positive-Control	Low-PBEH	Medium-PBEH	High-PBEH	Normal-Control
Final body weight (g)	454 ± 10.4 ^a^	447 ± 7.78 ^a^	458 ± 8.91 ^a^	434 ± 7.03 ^b^	429 ± 7.4 ^b^	453 ± 14 ^a^
Body weight gain (g)	178 ± 7.36 ^a^	171 ± 6.99 ^ab^	163 ± 6.83 ^b^	163 ± 4.44 ^b^	155 ± 6.34 ^c^	185 ± 11.4 ^a^
Visceral fat (%)	5.11 ± 0.65 ^a^	4.94 ± 0.44 ^ab^	4.74 ± 0.41 ^ab^	4.86 ± 0.55 ^ab^	4.2 ± 0.42 ^b^	5.19 ± 0.57 ^a^
Food efficiency	10.3 ± 0.42 ^b^	11.1 ± 0.64 ^a^	10.7 ± 0.33 ^ab^	9.81 ± 0.29 ^bc^	9.62 ± 0.32 ^c^	10.7 ± 0.39 ^ab^
Fasting serum glucose (mg/dL)	123 ± 3.7 ^b^	140 ± 6.7 ^a^	117 ± 4.74 ^b^	116 ± 5.78 ^b^	115 ± 3.55 ^b^	111 ± 2.36 ^b^
Fasting serum insulin (ng/mL)	0.58 ± 0.10 ^a^	0.47 ± 0.10 ^b^	0.56 ± 0.09 ^a^	0.55 ± 0.10 ^a^	0.51 ± 0.09 ^a^	0.27 ± 0.03 ^c^
HOMA-IR	6.12 ± 0.76 ^a^	5.85 ± 0.73 ^ab^	5.69 ± 0.87 ^ab^	5.57 ± 0.73 ^ab^	5.09 ± 0.71 ^b^	2.62 ± 0.18 ^b^

Food efficiency = weight gain/food intake; visceral fat = epididymal fat pads + retroperitoneal fat. HOMA-IR, homeostatic model assessment for insulin resistance. Male rats had intraperitoneal injections of scopolamine (2 mg/kg body weight/day) at 30 min after daily feeding of casein (MD-control), porcine brain enzyme hydrolysates (PBEH; 7 mg total nitrogen/mL) at 0.053 mL (Low-PBEH), 0.159 mL (Medium-PBEH), 0.478 mL (High-PBEH), or 10 mg donepezil (Positive-control) per kilogram body weight per day for six weeks. Values represent means ± standard deviations (*n* = 10); ^a–c^ Means on the same row with different superscript letters indicate a significant difference among the groups by Tukey test at *p* < 0.05.

**Table 3:** Proinflammatory cytokines and liver damage index in the circulation.

	MD-Control	Positive-Control	Low-PBEH	Medium-PBEH	High-PBEH	Normal-Control
Serum TNF-α (ng/mL)	0.67 ± 0.09 ^a^	0.43 ± 0.08 ^b^	0.63 ± 0.06 ^a^	0.55 ± 0.08 ^ab^	0.46 ± 0.07 ^c^	0.52 ± 0.04 ^b^
Serum IL-1β (pg/mL)	17.8 ± 2.14 ^a^	18.4 ± 2.16 ^a^	15.5 ± 2.51 ^a^	9.23 ± 1.76 ^b^	8.27 ± 1.29 ^b^	7.94 ± 1.02 ^b^
Serum ALT (U/L)	39.1 ± 3.41 ^a^	38.5 ± 3.23 ^a^	41.4 ± 3.39 ^a^	39.8 ± 2.48 ^a^	33.8 ± 2.83 ^b^	39.5 ± 2.78 ^a^
Serum AST (U/L)	21.6 ± 3.37 ^a^	24.5 ± 6.35 ^a^	21.5 ± 3.87 ^a^	23.3 ± 5.03 ^a^	13.8 ± 3.84 ^b^	19.4 ± 4.67 ^a^

PBEH, porcine brain enzyme hydrolysates; TG, triglyceride; ALT, alanine aminotransferase; AST, aspartate aminotransferase. TNF-α, tumor necrosis factor-α; IL-1β, interleukin-1β. Male rats had intraperitoneal injections of scopolamine (2 mg/kg body weight/day) at 30 min after daily feeding of casein (MD-control), porcine brain enzyme hydrolysates (PBEH; 7 mg total nitrogen/mL) at 0.053 mL (Low-PBEH), 0.159 mL (Medium-PBEH), 0.478 mL (High-PBEH), or 10 mg donepezil (Positive-control) per kilogram body weight per day for six weeks. Values represent means ± standard deviations (*n* = 10). ^a–c^ Means on the same row with different superscript letters indicate a significant difference among the groups by Tukey test at *p* < 0.05.

**Table 4:** Hippocampal lipids, oxidative stress, and neuroinflammation.

	MD-Control	Positive-Control	Low-PBEH	Medium-PBEH	High-PBEH	Normal-Control
Hippocampal triglyceride (mg/g)	47.5 ± 3.10	47.06 ± 1.75	47.4 ± 4.36	44.7 ± 3.66	46.2 ± 3.96	46.4 ± 2.45
Hippocampal cholesterol (mg/g)	18.0 ± 1.21 ^a^	15.0 ± 1.43 ^b^	15.5 ± 1.27 ^b^	13.6 ± 0.76 ^c^	14.5 ± 1.33 ^bc^	12.2 ± 0.82 ^d^
Hippocampal glycogen (mg/g liver)	30.5 ± 0.28 ^b^	31.5 ± 0.33 ^ab^	30.9 ± 0.36 ^b^	32.5 ± 0.21 ^a^	32.3 ± 0.42 ^a^	32.7 ± 0.38 ^a^
Hippocampal lipid peroxides (MDA μmol/g tissue)	3.85 ± 0.53 ^a^	2.94 ± 0.35 ^b^	3.35 ± 0.54 ^ab^	2.91 ± 0.47 ^b^	2.43 ± 0.44 ^bc^	2.31 ± 0.36 ^c^
Acetylcholinesterase activity (U/mg protein)	0.66 ± 0.09 ^a^	0.32 ± 0.07 ^d^	0.59 ± 0.07 ^ab^	0.53 ± 0.07 ^b^	0.41 ± 0.07 ^c^	0.12 ± 0.05 ^e^
Hippocampal *TNF-α* contents (ng/g tissue)	4.08 ± 0.26 ^a^	3.41 ± 0.19 ^b^	3.85 ± 0.27 ^ab^	3.43 ± 0.24 ^b^	3.44 ± 0.81 ^b^	3.20 ± 0.11 ^c^
Relative mRNA expression of hippocampal *TNF-α* (AU)	1 ^a^	0.88 ± 0.07 ^b^	0.97 ± 0.09 ^a^	0.91 ± 0.09 ^ab^	0.82 ± 0.09 ^bc^	0.78 ± 0.09 ^c^
Relative mRNA expression of hippocampal *IL-1β* (AU)	1 ^a^	0.94 ± 0.09 ^a^	1.01 ± 0.09 ^a^	0.92 ± 0.09 ^ab^	0.83 ± 0.07 ^b^	0.79 ± 0.08 ^b^
Relative mRNA expression of hippocampal *BDNF* (AU)	1 ^c^	1.51 ± 0.19 ^a^	1.07 ± 0.11 ^c^	1.25 ± 0.15 ^b^	1.43 ± 0.11 ^a^	1.36 ± 0.11 ^ab^
Relative mRNA expression of hippocampal *CNTF* (AU)	1 ^d^	1.51 ± 0.39 ^bc^	0.98 ± 0.24 ^d^	1.35 ± 0.29 ^c^	1.44 ± 0.21 ^bc^	3.13 ± 0.64 ^a^

Male rats had intraperitoneal injections of scopolamine (2 mg/kg body weight/day) at 30 min after daily feeding of casein (MD-control), porcine brain enzyme hydrolysates (PBEH; 7 mg total nitrogen/mL) at 0.053 mL (Low-PBEH), 0.159 mL (Medium-PBEH), 0.478 mL (High-PBEH), or 10 mg donepezil (Positive-control) per kilogram body weight per day for six weeks. MDA, malondialdehyde; *TNF-α*, tumor necrosis factor-α; *IL-1β*, interleukin-1β; *BDNF*, brain-derived neurotrophic factor; *CNTF*, ciliary neurotrophic factor. Values represent means ± standard deviations (*n* = 10). ^a–c^ Means on the same row with different superscript letters indicate a significant difference among the groups by Tukey test at *p* < 0.05.

## Data Availability

The data presented in this study are available upon request from the corresponding author.
